# Humoral and Innate Immunological Profile of Paediatric Recipients of Pfizer-BioNTech BNT162b2 mRNA Vaccine

**DOI:** 10.3390/microorganisms12071389

**Published:** 2024-07-09

**Authors:** Sundararaj Stanleyraj Jeremiah, Priya Das, Manu Venkatesan, Reem Albinzayed, Aysha Ahmed, Nigel John Stevenson, Martin Corbally, Manaf Alqahtani, Fatima Al-Wedaie, Eman Farid, Suha Hejres

**Affiliations:** 1 School of Postgraduate Studies and Research, Royal College of Surgeons in Ireland—Medical University of Bahrain, Building 2441, Road 2835, Busaiteen 228, Bahrain; pdas@rcsi.com (P.D.);; 2 Hematology and Hematopathology Laboratory, King Hamad University Hospital, Busaiteen 228, Bahrain; manu.venkatesan@khuh.org.bh (M.V.); suha.hejres@bdfmedical.org (S.H.); 3 Medical Internship, King Hamad University Hospital, Busaiteen 228, Bahrain; reem.albinzayed@khuh.org.bh (R.A.); aysha.ahmed@khuh.org.bh (A.A.); 4 Department of Surgery, Royal College of Surgeons in Ireland—Medical University of Bahrain, Busaiteen 228, Bahrain; martin.corbally@khuh.org.bh; 5 Department of Microbiology, Royal College of Surgeons in Ireland—Medical University of Bahrain, Busaiteen 228, Bahrain; mqahtani@rcsi-mub.com; 6 Department of Pathology, Salmaniya Medical Complex, Government Hospital, Manama 329, Bahrain; fwedaie@health.gov.bh (F.A.-W.); emanfarid57@gmail.com (E.F.); 7 Department of Microbiology, Immunology and Infectious Diseases, College of Medicine, Arabian Gulf University, Manama 329, Bahrain

**Keywords:** Pfizer vaccine, COVID-19, paediatric immunity, neutralizing antibodies, innate immunity, interferons

## Abstract

The Pfizer-BioNTech vaccine was one of the essential tools in curtailing the COVID-19 pandemic. Unlike conventional vaccines, this newly approved mRNA vaccine is taken up by cells, which leads to the synthesis of the specific viral Spike antigen. The vaccine was initially introduced for adults, and the immunological profile of adult recipients is well-characterized. The vaccine was approved for paediatric use much later after its efficacy and safety had been confirmed in children. However, the complete picture of how the paediatric immune system in children reacts to the vaccine is not well documented. Therefore, in order to better understand the immune response in children, we analysed the humoral response, immune cell count, and interferon signalling in paediatric vaccine recipients ranging between 5 and 17 years of age. Our findings suggest that the paediatric recipients elicit a robust humoral response that is sustained for at least three months. We also found that the vaccine triggered a transient lymphocytopenia similar to that observed during viral infection. Interestingly, we also found that the vaccine may sensitise the interferon signalling pathway, priming the cells to mount a potent response when exposed to interferons during a subsequent infection. The study offers new insights into the workings of the paediatric immune system and innate immunity, thereby opening the doors for further research in this field.

## 1. Introduction

The COVID-19 pandemic, which ravaged mankind, was effectively curtailed using mass vaccination [1]. Conventionally, vaccines comprised specific antigenic proteins along with adjuvants that elicit a neutralising antibody response against the vaccinated microbe. One of the salient aspects of the pandemic was the introduction of non-conventional mRNA vaccines. Upon vaccine administration, the mRNA is transcribed by the host cells to produce the defined viral antigen, which elicits a robust humoral and cellular response [2]. This not only mimics the biological scenario of viral infection but also produces antigenic proteins in vivo with post-translational changes similar to that happening during infection, making the mRNA vaccines more effective than conventional vaccines in the SARS-CoV-2-naïve population [3].

The efficacy of a conventional vaccine can be broadly assessed by quantifying the protective humoral response. Neutralising antibodies (nAbs) directed against the receptor binding domain (RBD) of the SARS-CoV-2 spike protein offer specific protection; however, the quantification of overall levels of anti-spike antibody (spAb) provides an arbitrary estimate of protection [4]. Also, since the vaccine has only the spike protein as the antigenic determinant, detection of antibodies other than SpAb, such as the anti-nucleocapsid protein antibody (NcpAb), denotes a history of SARS-CoV-2 infection. Apart from inducing humoral immunity, the BNT162b2 mRNA vaccine also elicits potent cell-mediated immunity comprising antigen-specific T-cell responses [5].

mRNA vaccines have also been reported to influence innate immunity. The innate immune system is the principal line of defence for viral infection, where the infected cells secrete type-1 interferons (IFN). Type-1 IFNs (α and β) bind to specific receptors, initiating a signalling cascade that activates several hundred IFN-stimulated genes (ISG), which are transcribed to produce various antiviral proteins and immunoreactive cytokines that modulate adaptive immunity [6]. Among the several ISG proteins, ISG-15 is the central player regulating viral replication through both non-covalent binding to viral proteins and its action as a cytokine [7]. SARS-CoV-2 has been reported to evade or dampen IFN signalling to promote its survival [8]. Since the BNT162b2 mRNA vaccine triggers the presentation of spike antigens, it essentially harnesses innate immunity. However, there is looming controversy regarding the effect of the mRNA vaccine on innate immunity, as certain studies claim that it impairs type-1 IFN signalling [9], while others report that type-1 IFN response is potentiated by the vaccine [10].

The Pfizer-BioNTech BNT162b2 mRNA vaccine was initially approved only for adults, and it has shown considerable efficacy over the years despite the evolution of SARS-CoV-2 variants [11]. Later, it was approved for children over six months, and studies showed good protective efficacy in children immunised with two doses. Studies showed that the vaccine efficacy (VE) varies with age and is higher in older children (above 12 years) compared to younger children [12]. VE is also reported to wane over time in younger children, falling to less than 50% in two months following the receipt of the second dose [13]. In children, infection-induced nAbs were found to last for 16 months [14], whereas the nAbs induced upon vaccination with an inactivated vaccine lasted for over three months [15]. 

With this background, we assessed the immunological profile of children vaccinated with two doses of Pfizer-BioNTech BNT162b2 mRNA vaccine, following up the immunological parameters until 90 days post-vaccination. We also assessed the levels of vaccine-derived spike antibodies and their sustenance over time, along with changes in immune cell counts during the course of vaccination and detected whether the vaccine influences the functioning of innate immunity with respect to IFN signalling. We found that paediatric vaccine recipients elicit a robust humoral response that is sustained for at least three months. The vaccine triggered a transient lymphocytopenia similar to that observed during viral infection. Interestingly, we observed that the vaccine may sensitise the interferon signalling pathway, priming the cells to mount a potent response when exposed to interferons during a subsequent infection.

## 2. Methods

### 2.1. Study Design

A prospective cohort design was adopted wherein two groups of paediatric population (12–17 years and 5–11 years) were eligible for participation in the study. The study was conducted after the National Medical Taskforce for COVID-19 in Bahrain approved Pfizer vaccination among children. Ethical approval of the study was obtained from the COVID-19 Taskforce in Bahrain and the King Hamad University Hospital Institutional Review Board.

### 2.2. Screening and Recruitment of Participants

The vaccination dates of the children were charted by the COVID-19 task force. Participants were enrolled between November 2021 to April 2022. The inclusion criteria were to include children from ages 5–11 and 12–17 who came to receive vaccinations. Parents and the children were seen by the investigators prior to vaccination, and the research protocol was explained in detail. Children with a known diagnosis of an immunocompromised condition or on treatment with immunosuppressive therapy were excluded from the study. After obtaining written consent from the parents, a questionnaire was filled out by the parents to collect the general demographics and clinical history of the children recruited for the study. All participants received two doses of the BNT162b2 vaccine as per the manufacturer’s guidelines.

### 2.3. Blood Sample Collection

Blood samples were collected at three time points: 1. Prior to first dose vaccination (Day 0), 2. Prior to the second dose of vaccination (Day 21), and 3. Three months after administering the first dose of vaccine (Day 90). Up to 20 mL of venous blood was collected from the cubital vein into EDTA and serum separator tubes for FACS analysis, peripheral blood mononuclear cells (PBMC) isolation and harvesting of serum, which were all performed within 24 h. Harvested sera and PBMCs were stored at −80 °C until further analysis.

### 2.4. Immune Cell Counts by FACS

Flow cytometric evaluation was performed on Beckman Coulter Navios EX three laser flow cytometer, and analysis was conducted on Kaluza C software version 1.1. The flow cytometry assessment on 100 microliters whole blood EDTA sample was conducted using a single platform, no wash lyse technique. Two panels were designed using Beckman Coulter reagents and Flow-Count Fluorospheres to evaluate and quantify the WBCs and dendritic cells (Supplementary Table S1). All WBCs (Neutrophils (CD16+), B lymphocytes (CD19+), NK cells (CD 56 +/CD3−), T helper (CD 4+/CD3+) and cytotoxic T cells (CD8+/CD3+)) were evaluated using tube 1. Percentage distribution and absolute counts were tabulated for all cases (Supplementary Figure S1). Monocytes and Dendritic cells were further evaluated in the second tube. The approach briefly was: in the CD45 vs LIN gate, lineage-negative cells (i.e., non-CD3, CD19, CD20, CD56) were gated and further analysed on HLADR vs. CD14 gate to isolate the monocytes (CD14+/HLADR+/LIN−). HLADR positive, non-monocytic-non-NK (HLADR+/CD14−/CD56−) dendritic cells were further analysed on CD123 versus CD11c gate to enumerate pDCs (CD123+/CD11c−) and mDCs(CD123−/CD11c+). DC Cells that expressed partial CD123 and CD11c were categorised as others DC. (Supplementary Figure S2) Percentage distribution and absolute counts were tabulated. Basophils (CD123+/HLADR−) in the sample acted as internal controls.

### 2.5. ELISA for Anti-Nucleocapsid Protein Antibody of SARS-CoV-2

Anti-NcpAb IgG ELISA (Abbexa, Cambridge, UK) was performed on Day 0 serum samples to detect the IgG antibodies against the SARS-CoV-2 Nucleocapsid protein to confirm COVID-19 infection prior to vaccination. The ELISA plate provided in this kit comprised wells pre-coated with purified SARS-CoV-2 Nucleocapsid protein and detected Anti-NcpAb IgG by the indirect ELISA principle. The test was performed according to the manufacturer’s instructions, and the optical density (OD) was measured at a wavelength of 450 nm using a Multiskan SkyHigh Microplate Spectrophotometer (Thermo Fischer, Franklin, MA, USA). The absorbance value of each test sample was compared with the cut-off value to judge the presence or absence of SARS-CoV-2 Anti-NcpAb.

### 2.6. Chemiluminescence Assay for Anti-Spike Protein Antibody of SARS-CoV-2

Anti-S AdviseDx SARS-CoV-2 IgG II Qaunt chemiluminescence assay (Abbott, Chicago, IL, USA) was used to detect the anti-SpAb in sera obtained on Day 0, Day 21, and Day 90 from each participant. The SARS-CoV-2 spike antigen-coated paramagnetic microparticles bound to the anti-SpAbs to emit chemiluminescence upon the addition of anti-human labelled IgG. The test provided automated quantitative results and was considered positive if the value was above 50 AU/mL. The upper limit of detection of the assay was 500 AU/mL.

### 2.7. PBMC Isolation

PBMCs were isolated from EDTA blood samples within 24 h of collection using Ficoll^®^ Paque Plus (Cytiva, Marlborough, MA, USA) density gradient centrifugation. The cells were resuspended at a count of 5 × 10^6^ per ml in a freeze-stock medium comprising 90% fetal calf serum (FCS) and 10% DMSO. The cell suspension was aliquoted one ml each into screw-capped tubes and frozen in Nalgene^®^ Mr. Frosty (Sigma-Aldrich, St. Louis, MO, USA) with isopropanol at –80 °C and stored until further analysis.

### 2.8. IFNα Challenge Assay- qPCR for ISG

Frozen PBMCs were revived as described previously to ensure maximal viability [16]. The cells were thawed by immersing in a 37 °C water bath and quickly suspended in 10 mL of prewarmed RPMI containing 20% FCS in a 15 mL Falcon tube. The cells were centrifuged at 500× *g* for 10 min, and the supernatant was removed to wash off the excess DMSO. The cells were resuspended in RPMI with 10% FCS, and 5 × 10^4^ viable cells were seeded per well in a 24-well plate and incubated at 37 °C for 3 h. 10 ng/mL IFN-2α (Sigma-Aldrich, St. Louis, MO, USA) was added to each well and incubated for 4 h to allow the expression of ISGs [17]. Cellular RNA was extracted using RNeasy Plus Mini Kit (Qiagen, Venlo, The Netherlands) and reverse transcribed using QuantiTect Reverse Transcription Kit (Qiagen, Venlo, The Netherlands). The cDNA master mix was prepared using QuantiNova SYBR Green PCR Kit (Qiagen, Venlo, The Netherlands) with the ISG-15 primer pair (Forward: 5′ TCCTGCTGGTGGTGGACAA3′ and Reverse: 3′TTGTTATTCCTCACCAGGAT5′) in Applied Biosystems 7500 Real-Time PCR System (Applied Biosystems, Waltham, MA, USA). Fold change of MxA expression was calculated after normalising expression over the housekeeping gene β-Actin (Forward: 5′GGACTTCGAGCAAGAGATGG3′ and Reverse: 3′AGCACTGTGTAGCCGTACAG5′).

### 2.9. Statistical Methods

Spike antibody data was log-transformed to attain normal distributions. Repeated measures ANOVAs and Mann–Whitney tests were used to assess the statistical significance of the differences in the three different time periods. Wilcoxon signed-rank test for used to test the differences in spike antibody levels in previously infected with COVID-19 and non-infected groups over the different time points. Differences in the immune cells’ numbers among the infected and non-infected groups were also assessed using the Wilcoxon signed-rank test. Statistical analysis was conducted using IBM SPSS Statistics 26.0(US), and visualisations were generated using GraphPad Prism version 10.0 (GraphPad Software, San Diego, CA, USA).

## 3. Results

### 3.1. Study Participants and Demographics

A total of 42 children were screened as per the inclusion criteria, 15 in the 5–11 years cohort and 27 in the 12–17 years cohort, who all completed two doses (Day 0 and Day 21) of the vaccination Pfizer-BioNTech BNT162b2 mRNA vaccine. However, six children from the former and two from the latter cohorts were lost during follow-up at Day 90 and hence were excluded from the study. Table 1 shows the demographics of the final participants, 9 and 25 children in 5–11- and 12–17-years cohorts, respectively.

### 3.2. Anti Spike Antibody Levels

**Figure 1:**
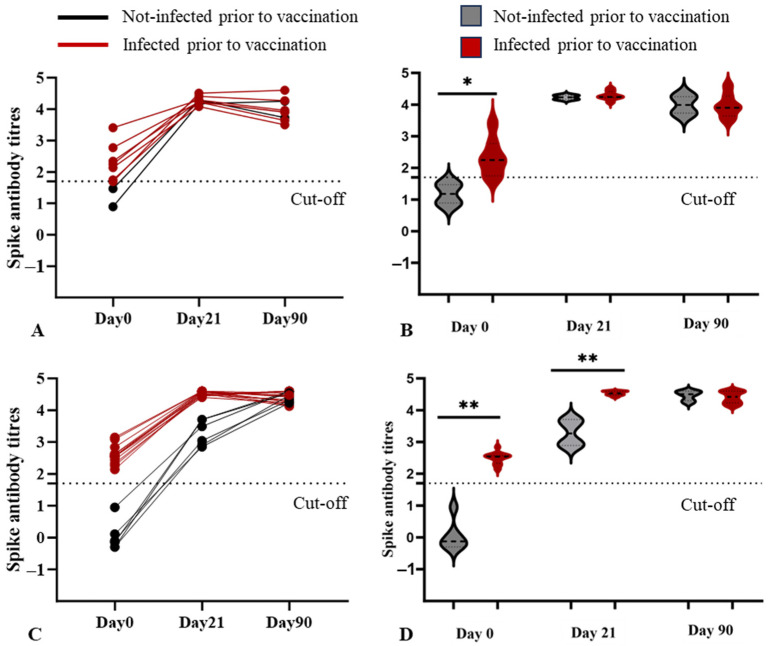
Anti Spike antibody levels (**A**,**B**) 5–11-years cohort (**C**,**D**) 12–17-years cohort. Values above the cut-off are considered positive and below negative. Panels (**A**,**C**) represent individual responses, and panels (**B**,**D**) represent distribution per group. The presence or absence of infection was determined by the Anti-Nucleocapsid protein antibody. Units converted to log scale. ** indicates *p* < 0.001 and * indicates *p* < 0.05.

In both 5–11- and 12–17-years cohorts, Day 0 samples were positive (above the cut-off) for SpAb in the previously infected and were negative (below the cut-off) in the uninfected (Figure 1). Following the first vaccine dose, SpAb levels increased on Day 21 in both previously infected and uninfected subsets of both cohorts. The previously infected subsets of both cohorts reached peak detectable levels of SpAb after the first dose (Figure 1A,C). However, the uninfected subset of the 5–11 years cohort achieved peak levels with the first dose, similar to the trend elicited by the previously infected (Figure 1A,B). The uninfected subset of the 12–17 years cohort showed a significant rise in SpAb levels compared to Day 0, but the levels were significantly lower than peak levels achieved by the infected subset at this time point (Figure 1D). SpAb levels sustained high levels in all samples at Day 90. Although the Day 90 levels showed a slight reduction compared to Day 21 in the 5–11-years cohort, this difference was not statistically significant. 

### 3.3. Immune Cell Counts

**Figure 2:**
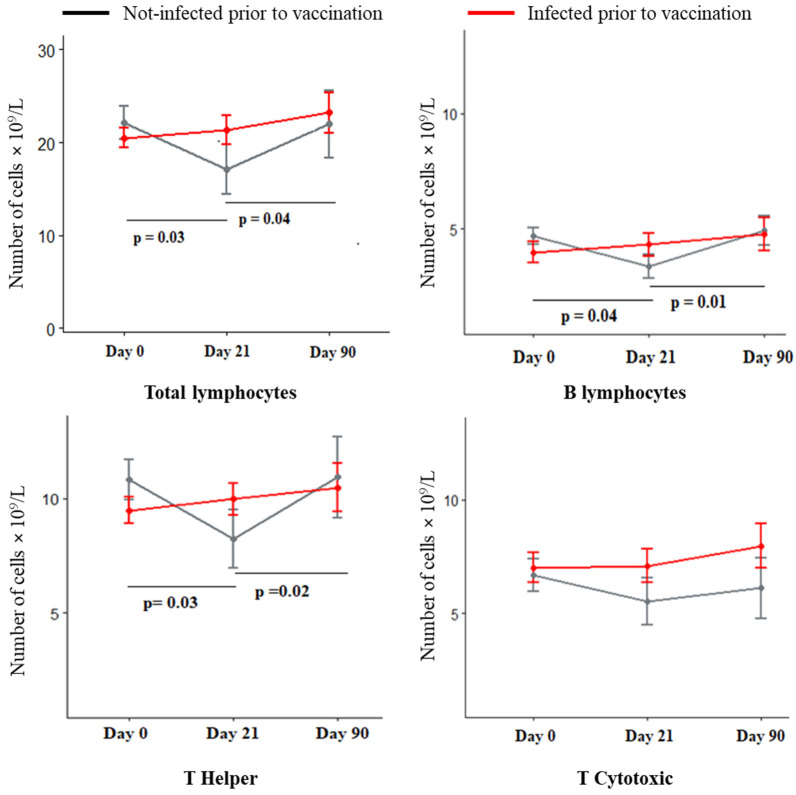
Absolute cell counts of lymphocyte populations. Lymphocyte counts at the three-time points were assessed by flow cytometry. Mean ± SE, *p* < 0.05, represents a significant change in counts.

All immune cell populations were quantified by flow cytometry during the three-time points (Supplementary Figure S3). Populations of most of the immune cells did not show any fluctuation over the vaccination course, except for lymphocytes (Figure 2). The first dose of vaccination elicited a significant drop in lymphocyte counts, which increased after the second dose and was found to be at the basal level on the 90th-day samples. This pattern resembles the acute transient lymphopenia that occurs during viral infection and is markedly observed with B lymphocytes and T-helper cells [18].

### 3.4. Interferon-Stimulated Gene Expression to Type-1 Interferon Challenge

**Figure 3:**
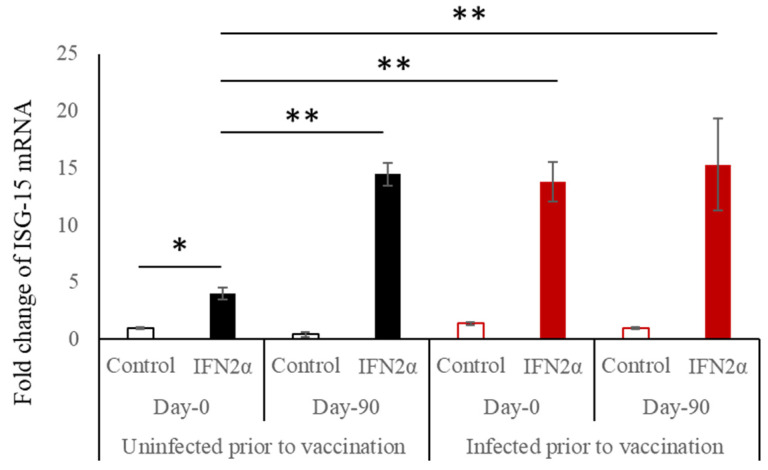
ISG-15 expression upon IFN2α challenge. The mean fold change of ISG-15 mRNA was observed in untreated controls and PBMCs treated with IFN2α for four hours. The data depicted is the mean of experiments conducted in duplicates. ** indicates *p* < 0.001 and * indicates *p* < 0.05.

Type-1 IFNs initiate the IFN signalling cascade to express several hundreds of ISGs. ISG-15 is one of the most abundant ISGs produced in the SARS-CoV-2 infection [19]. Treatment of cells in vitro with type-1 IFNs has been shown to increase the expression of ISG mRNA in 4 h [17]. We challenged the PBMCs collected from the participants with IFN2α and analysed the expression of ISG-15 (Figure 3). ISG-15 was expressed upon IFN stimulation in all subsets tests, and there was no difference between the 5–11 and 12–17 cohorts. In the uninfected Day 0 samples, the naïve PBMCs which were not exposed to vaccine or infection showed a modest increase of ISG-15. The uninfected Day 90 samples expressed significantly more ISG-15 than Day 0, suggesting that the vaccine-exposed mononuclear cells are primed/sensitised to secrete more ISG upon IFN stimulation. All infected samples (Day 0 and Day 90) showed a significant increase in ISG-15, suggesting that the infection causes this sensitisation with or without vaccine influence.

## 4. Discussion

In about a year after the outbreak of the COVID-19 pandemic, the Pfizer-BioNTech BNT162b2 mRNA vaccine was first approved for immunising adults [20]. Following its success in adults, it was later approved for children above six months of age [21]. Studies show long-term sustenance of vaccine-derived antibodies in adults for over 12 months following infection [22,23], and they were found to wane around 6 months after the second dose [24]. Breakthrough infections and boosters enhance the protection in these instances [24,25]. Studies on the sustenance of nAb levels in children are not available. However, an assessment of vaccine efficacy (VE) based on hospitalisations by Klein et al. has shown the VE in the 12–17-years cohort to be around 80% before Day 150 and in the 5–11-years cohort, VE was only 46% before Day 67 [13]. Fowlkes et al. have reported the VE at Day 150 to be 59% in the 12–17-years cohort and 31% in the 5–11-years cohort. Although both the above reports show differences in VE between the younger and older cohorts, it could possibly be because the vaccine was approved earlier for the 12–17 cohort prior to the onset of Omicron, while the 5–11-years cohort would have been tested later while the Omicron strain was rampant. We ruled out the possibility of the above bias by collecting samples during the same timeframe of Omicron prevalence and observed sustained high levels of antibodies on Day 90 in both cohorts. However, the antibody levels might not reflect the actual VE conferred, as the vaccine-induced SpAbs might vary in their efficiency in neutralising the Omicron and the other newer variants of SARS-CoV-2. 

Viral infections cause transient lymphocytopenia due to various mechanisms [18]. We observed a similar effect with vaccination, especially with B lymphocytes and T helper cells, which reverted to basal counts after vaccination. Although leukopenia is one of the documented yet less frequent adverse effects of mRNA vaccines [26], this transient phenomenon could possibly be due to the action of mRNA vaccine mimicking viral infection. This should be taken into consideration during vaccination while the recipient may be on treatment with other drugs that can concomitantly cause lymphocytopenia [27].

Dendritic cells (DCs) are a part of the innate and adaptive immune system, where they play the roles of both regulators and effectors. Blood DCs comprise multiple subsets which, the main ones being plasmacytoid DCs (pDCs) and myeloid DCs (mDC). pDCs are specialised in producing a rapid type-1 IFN response to viral infections [28], while mDCs perform the classical functions of DCs in antigen presentation on HLA class II molecules [29]. The counts of DC subpopulations show an age-dependent variation in healthy children, and there is much less literature examining the variation in DC counts of children during infection. The range of DCs observed in our study in the uninfected Day 0 cohort matched with the reference range of DCs of healthy children reported in the literature (Supplementary Figure S3) [30]. Hence, we intended to assess if vaccination induced any changes in DC counts. However, the sample volume and size were too low to pick up meaningful variation of the sparsely populated DCs.

There have been claims of innate immune suppression by SARS-CoV-2 mRNA vaccinations, especially that vaccination impairs the type-1 IFN signalling [9]. SARS-CoV-2 uses various proteins to evade innate immunity [8], and the spike protein has been reported to restrict both the production and signalling of type-1 IFN [31,32]. Li et al. have shown evidence that the vaccine enhances innate responses after secondary immunisation. They have shown this in the acute phase, on Day 1, following secondary immunisation [10]. We designed an IFN challenge assay to see if there is any long-term effect of infection or vaccination (at Day 90) on innate immunity. This was under the rationale that viral reinfection will induce IFN secretion, and we intended to assess whether there is any impairment in IFN signalling, as claimed by other reports [9].

ISG-15 is one of the most abundant ISGs, and SARS-CoV-2 infection is known to cause profuse secretion of ISG-15 [19]. It acts by binding to various host and viral proteins both in the extracellular and intracellular milieu and SARS-CoV-2 evades ISG-15 action using its papain-like protease (PLpro) to dissociate bound ISG-15 from viral proteins [33]. Due to the above reasons, we chose ISG-15, among the hundreds of other ISGs produced. Since ISG-15 is predominantly secreted by mononuclear cells and macrophages, we chose to assess them in PBMCs [34]. Interestingly, we observed increased ISG-15 expression following IFN challenge in both the vaccinated and infected, suggesting that the vaccine does not affect its secretion. Robust ISG-15 elevation upon IFN challenge was observed at Day 90 compared to Day 0 in the uninfected, suggesting that this can be caused by either the vaccine or breakthrough infections. The findings suggest the possibility of a sensitisation or a memory phenomenon in IFN signalling that is primed by an earlier stimulus provided by a viral infection which is not dampened by the vaccine. This phenomenon warrants to be proven conclusively using further studies.

The duration of the study was too short to observe the decline of SpAb levels. We could not observe long-term sustenance since we ended the study on Day 90 due to increased dropouts on Day 180. Following up at 180 days would have been interesting to see if there was indeed any significant reduction in SpAb levels and if there were any differences between the two cohorts. Also, we used SpAb levels as a blanket marker to assess protection, as the anti-RBD antibodies are included in it. Despite lacking specificity, SpAb IgG/A/M titers have been shown to have a positive correlation with nAb titers [4,35,36]. Although we observed a robust increase in SpAb, their ability to confer specific protection against different variants of SARS-CoV-2 was not studied. This is especially important with the increased circulation of the Omicron variant, which has a high propensity for vaccine escape [37]. Ideally, the gold standard live virus neutralisation test could have provided information on mutant-specific inhibition of vaccine-derived antibodies, but we were unable to perform that in our biosafety level-2 facility. We had identified the presence or absence of infection only on Day 0 by detecting NcpAb. Breakthrough infections could have happened in either of the cohorts at any time until the Day 90 endpoint. Since we did not rule out this possibility with a confirmatory test, we could not conclusively suggest whether the persistence of SpAb at Day 90 was indeed due to vaccine or breakthrough infections. Anti-Ncp IgG detection is the widely used and the only practically available method to prove previous SARS-CoV-2 infections. However, since the anti-Ncp antibodies decline over time, the accuracy of this test in detecting remote infections earlier than 12 months is questionable. As this is an inherent fallacy of the only practically available testing method, we ruled out past infection based on both history and anti-Ncp IgG detection ELISA. None of the individuals in the uninfected cohort had a history of signs of COVID-19 in the previous year, and they were not exposed to people who had confirmed COVID-19. These subjects not only had a negative result with anti-Ncp IgG ELISA but also did not show a rapid rise of SpAb with the first vaccine dose, which is representative of a secondary humoral response. Hence, we could say with a high degree of certainty that the subjects of the previously uninfected cohort were indeed not previously infected with SARS-CoV-2. The time points of sample collection were suited more for studying the humoral response but were too broad for identifying specific fluctuations of immune cell counts. Finally, the sample size was low due to attrition over time, which hindered the assessment of cell counts of sparse cell types.

## 5. Conclusions

Despite the fallacies, the study provides some valid findings. In paediatric recipients over five years of age, two doses of the Pfizer-BioNTech BNT162b2 mRNA vaccine elicit a robust humoral response that is sustained for at least 90 days after the first dose of vaccination. The surge of ISG-15 upon IFN stimulation in the vaccinated suggests that it is unlikely that the vaccine dampens ISG-15 expression. However, the possibility of vaccine-induced suppression of IFN secretion and inhibition of other ISGs should be evaluated in further studies.

## Figures and Tables

**Table 1:** Demographics of the study participants.

Demographics		5–11 Years	12–17 Years
Gender	Male	5	15
	Female	4	10
Day 0 NP ELISA (Infection status)	Positive (Previously infected)	7	15
	Negative (Uninfected)	2	10
Comorbidities	Yes	-	2 (T1DM)
	No	9	23

## Data Availability

All data generated or analysed during this study are included in this published article.

## Supplementary Materials

The following supporting information can be downloaded at: https://www.mdpi.com/article/10.3390/microorganisms12071389/s1, Table S1: List of antibodies and panel design used to evaluate WBC and Dendritic cells by flowcytometry; Figure S1: Gating strategy for identification of WBCs; Figure S2: Gating strategy for identification of plasmacytoid DCs and myeloid DCs; Figure S3: Analysis of immune cell counts over time.
