# Supplementary material for: Humoral and Innate Immunological Profile of Paediatric Recipients of Pfizer-BioNTech BNT162b2 mRNA Vaccine
Source: Microorganisms. 2024 Jul 9;12(7):1389. doi: 10.3390/microorganisms12071389 (PMC11278604; doi:10.3390/microorganisms12071389)
Supplement: Supplementary file 1 [file microorganisms-12-01389-s001.zip › microorganisms-3064321-supplementary.pdf]

Supplementary material

Supplementary Table S1. List of antibodies (Beckman Coulter) and panel design used to evaluate WBC and Dendritic cells by flowcytometry.

**Antibody Panel for flowcytometry**										
	FL1	FL2	FL3	FL4	FL5	FL6	FL7	FL8	FL9	FL10
**TUBE/ AB**	FITC	PE	ECD	PC5.5	PC7	APC	APC 700	APC 750	PB	KO
**Tube 1**		CD56	CD19		CD8	CD3	CD16		CD4	CD45
**CAT. NO**		A07788	A07770		737661	IM2467	B20023		B49197	B36294
**CLONE**		N901	HD237		SFCI21 Thy2D3	UCHT1	3G8		13B8.2	J33
**Tube 2**		Lineage Cocktail	CD123	CD56		CD14	CD11c		HLA-DR	CD45
**CAT. NO**		B29559	B20027	B49189		IM2580U	B43304		B36291	B36294
**CLONE**			SSDCLY 107D2	N901		RMO52	BU15		Immu-357	J33

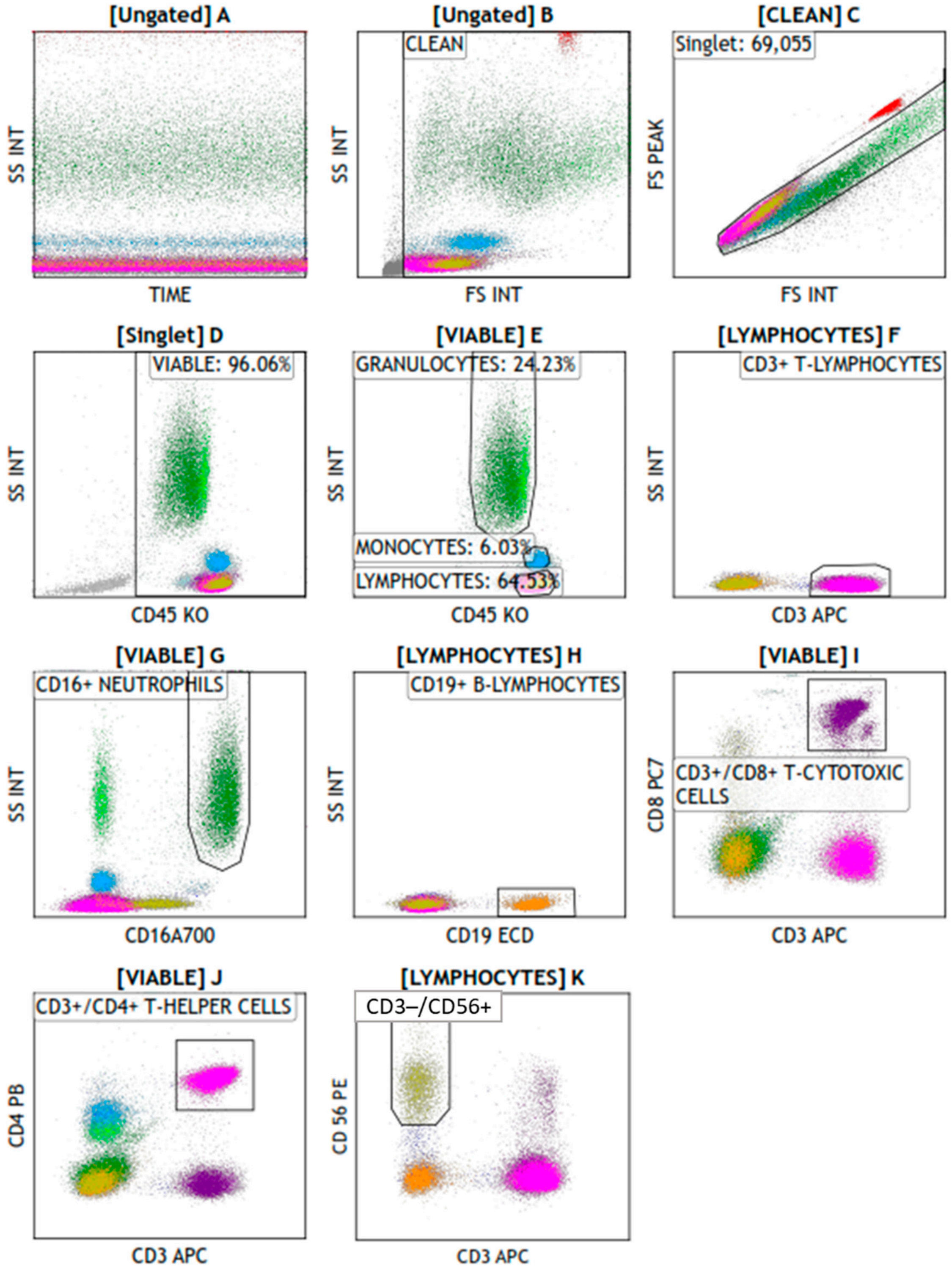

[Ungated] A
SS INT
TIME
[Ungated] B
CLEAN
SS INT
FS INT
[CLEAN] C
Singlet: 69,055
FS PEAK
FS INT
[Singlet] D
VIABLE: 96.06%
SS INT
CD45 KO
[VIABLE] E
GRANULOCYTES: 24.23%
MONOCYTES: 6.03%
LYMPHOCYTES: 64.53%
SS INT
CD45 KO
[LYMPHOCYTES] F
CD3+ T-LYMPHOCYTES
SS INT
CD3 APC
[VIABLE] G
CD16+ NEUTROPHILS
SS INT
CD16A700
[LYMPHOCYTES] H
CD19+ B-LYMPHOCYTES
SS INT
CD19 ECD
[VIABLE] I
CD3+/CD8+ T-CYTOTOXIC CELLS
CD8 PC7
CD3 APC
[VIABLE] J
CD3+/CD4+ T-HELPER CELLS
CD4 PB
CD3 APC
[LYMPHOCYTES] K
CD3–/CD56+
CD 56 PE
CD3 APC

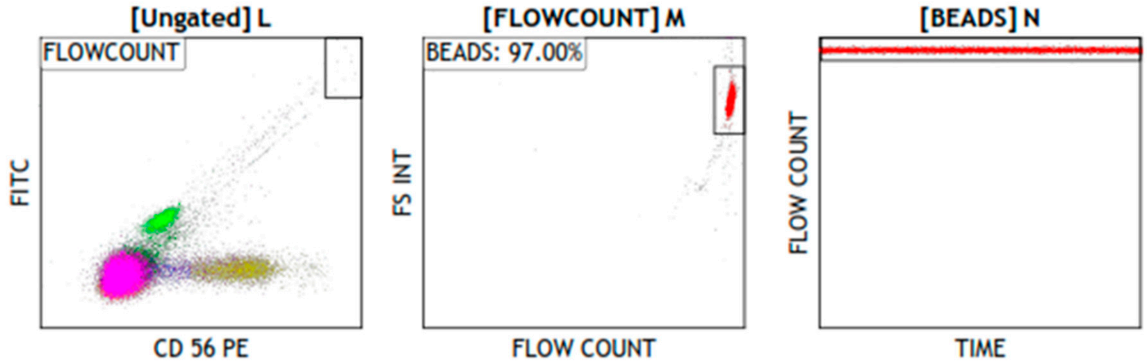

Gate	%Gated	Cells/µL
BEADS	97.00	1,007
VIABLE	96.06	4,711
CD16+ NEUTROPHILS	19.27	908
CD3+/CD4+ T-HELPER CELLS	27.09	1,276
CD3+/CD8+ T-CYTOTOXIC CELLS	16.98	800
LYMPHOCYTES	64.53	3,040
CD19+ B-LYMPHOCYTES	13.97	425
CD3–/CD56+	10.46	318

Supplementary Figure S1. Gating strategy for identification of WBCs. (A) time plot. (B) Region is set to identify leucocytes via granularity (SS) and cell size (FS) Smaller cells are excluded. (C) Regional gate (SINGLET) is used to exclude doublets and beads based on FS peak and FS (int). (D) Region VIABLE is set to include all CD45+ events (leucocytes). (E) Neutrophils, monocytes and lymphocytes are gated based on CD45 and side scatter (SS) parameters. (F) T-cells are identified using CD3 gating. (G) Neutrophils are enumerated based on CD 16 expression. (H) B-lymphocytes are identified based on CD 19 expression. (I) Cytotoxic T-cells are identified based on co-expression of CD 3 and CD 8. (J) Helper T-cells are identified using co-expression of CD 3 and CD4. (K) Natural Killer (NK) cells are identified by positive expression of CD 56 while being negative for CD 3. (L, M, N) gating flow-count fluorospheres.

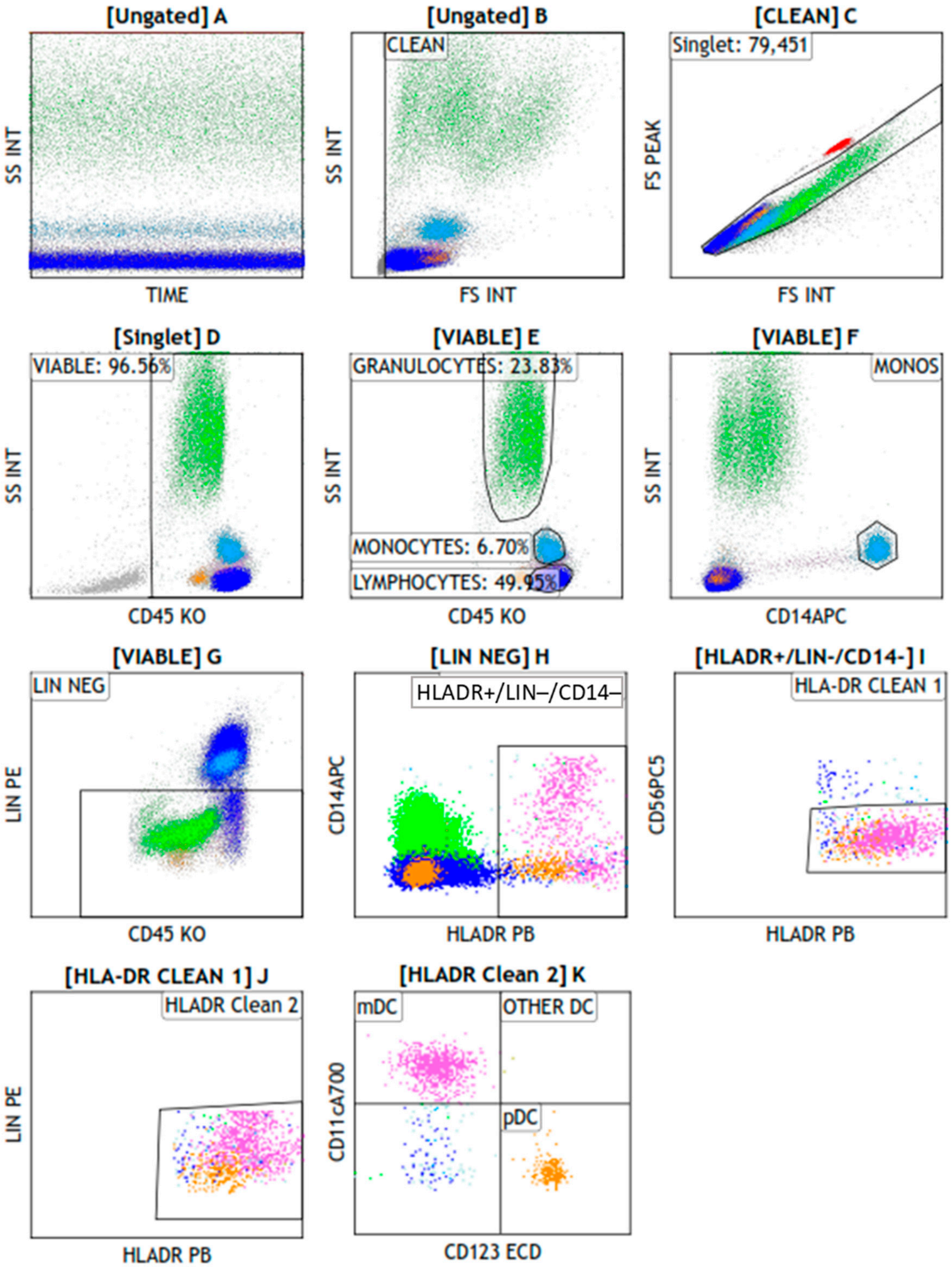

[Ungated] A
SS INT
TIME
[Ungated] B
CLEAN
SS INT
FS INT
[CLEAN] C
Singlet: 79,451
FS PEAK
FS INT
[Singlet] D
VIABLE: 96.56%
SS INT
CD45 KO
[VIABLE] E
GRANULOCYTES: 23.83%
MONOCYTES: 6.70%
LYMPHOCYTES: 49.95%
SS INT
CD45 KO
[VIABLE] F
MONOS
SS INT
CD14APC
[VIABLE] G
LIN NEG
LIN PE
CD45 KO
[LIN NEG] H
HLADR+/LIN–/CD14–
CD14APC
HLADR PB
[HLADR+/LIN-/CD14-] I
HLA-DR CLEAN 1
CD56PC5
HLADR PB
[HLA-DR CLEAN 1] J
HLADR Clean 2
LIN PE
HLADR PB
[HLADR Clean 2] K
mDC
OTHER DC
pDC
CD11cA700
CD123 ECD

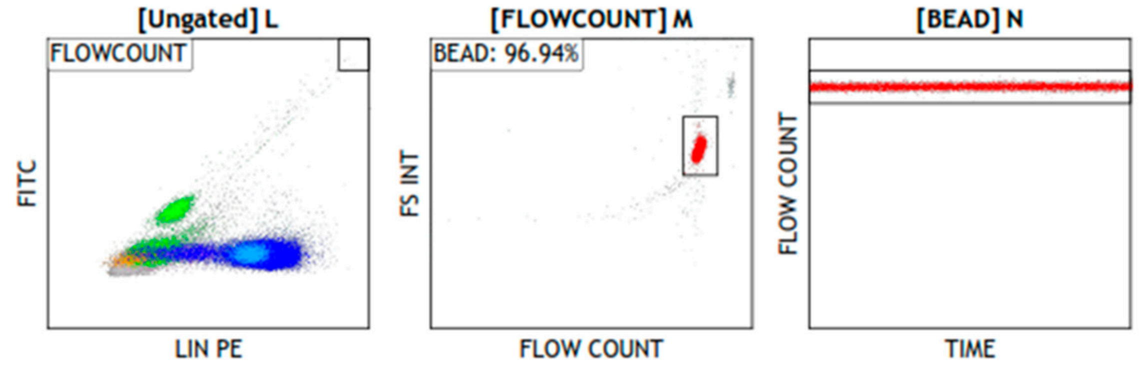

Gate	%Gated	Cells/µL
BEAD	96.94	1,007
VIABLE	96.56	4,659
LIN NEG	34.88	1,625
HLADR+/LIN–/CD14–	4.57	74
HLA-DR CLEAN 1	93.95	70
HLADR Clean 2	100.00	70
mDC	65.97	46
OTHER DC	0.17	0
pDC	21.76	15
MONOS	6.13	286

Supplementary Figure S2. Gating strategy for identification of plasmacytoid DCs (pDCs) and myeloid DCs (mDC). (A) time plot. (B) Region is set to identify leucocytes via granularity (SS) and cell size (FS) Smaller cells are excluded. (C) Regional gate (SINGLET) is used to exclude doublets and beads based on FS peak and FS (int). (D) Region VIABLE is set to include all CD45+ events (leucocytes). (E) Neutrophils, monocytes and lymphocytes are gated based on CD45 and side scatter (SS) parameters. (F) Monocytes are evaluated using CD14 gating. (G) Lineage (LIN) negative cells were isolated using a lineage cocktail (CD 19, CD20, CD 56, CD3). (H) LIN negative non monocytes that are HLADR positive are identified based on CD 14 and HLADR expression. (I &J) The target cells are further cleaned up to exclude contaminating cells. (K) HLADR positive, non-monocytic-non-NK (HLADR+/CD14–/CD56) dendritic cells were further analyzed on CD 123 versus CD11c gate to enumerate pDCs (CD123+/CD11c–) and mDCs (CD123–/CD11c+). DC Cells that expressed partial CD 123 and CD 11c were categorized as others DC (L, M, N) gating flow-count fluorospheres.

12-17 years	5-11 years
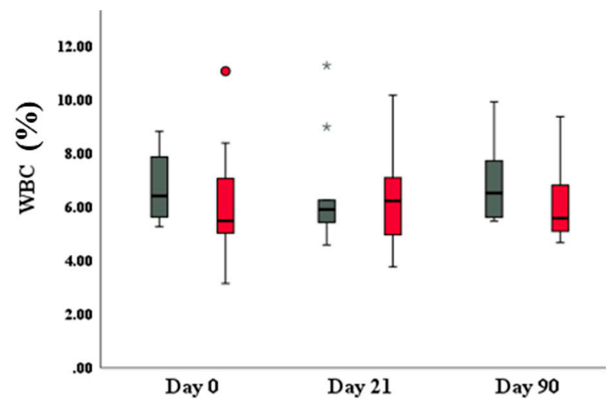	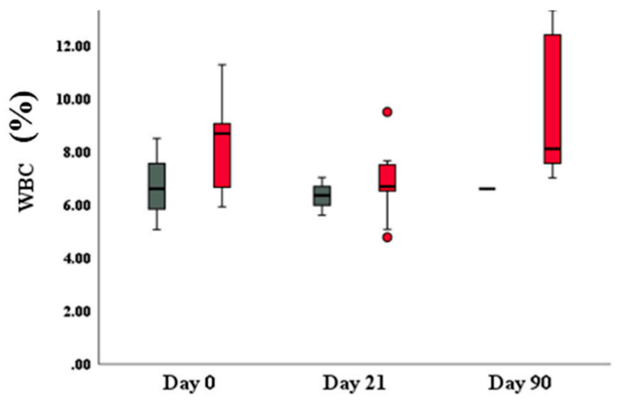
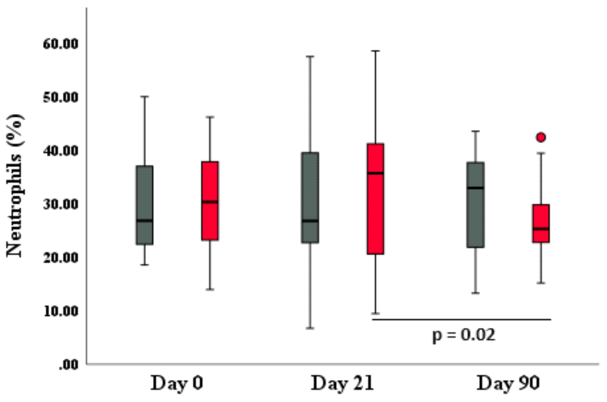	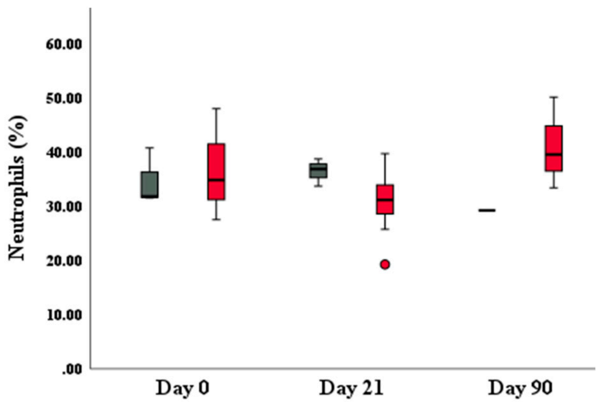
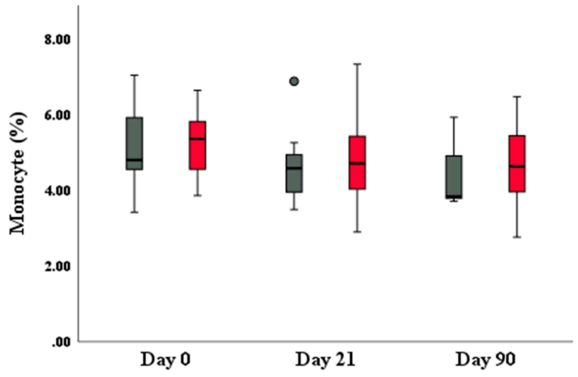	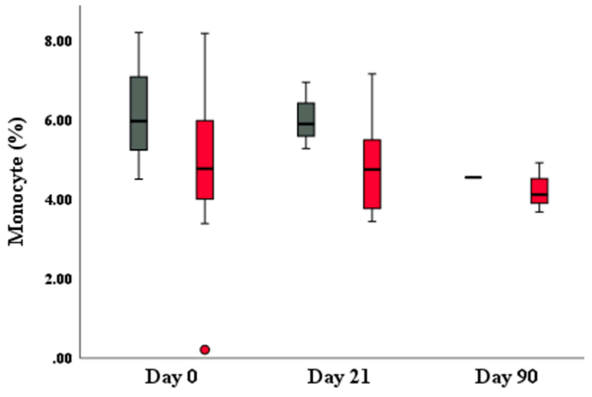

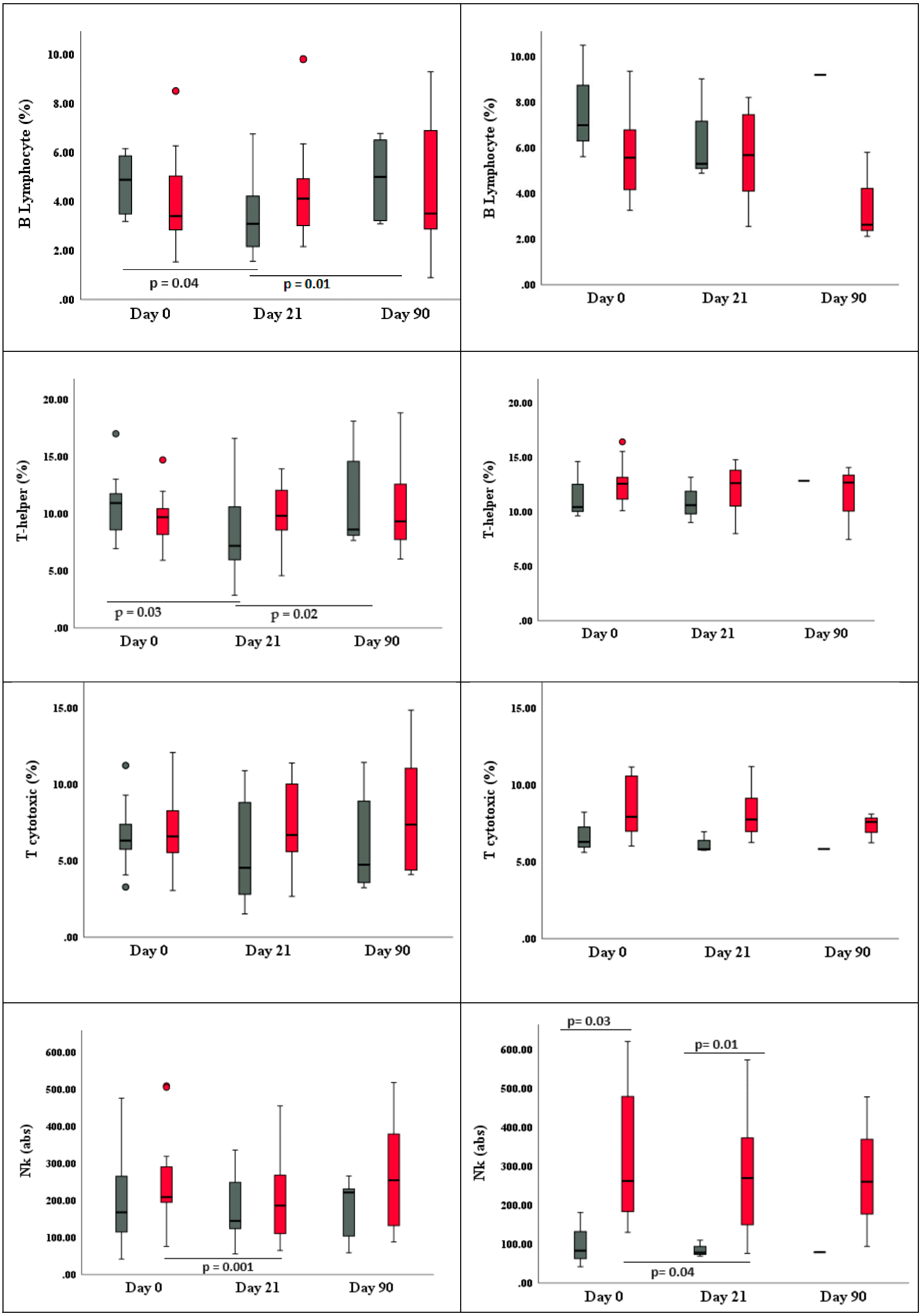
B Lymphocyte (%)
10.00
8.00
6.00
4.00
2.00
.00
p = 0.04
p = 0.01
Day 0
Day 21
Day 90
B Lymphocyte (%)
10.00
8.00
6.00
4.00
2.00
.00
Day 0
Day 21
Day 90
T-helper (%)
20.00
15.00
10.00
5.00
.00
p = 0.03
p = 0.02
Day 0
Day 21
Day 90
T-helper (%)
20.00
15.00
10.00
5.00
.00
Day 0
Day 21
Day 90
T cytotoxic (%)
15.00
10.00
5.00
.00
Day 0
Day 21
Day 90
T cytotoxic (%)
15.00
10.00
5.00
.00
Day 0
Day 21
Day 90
Nk (abs)
600.00
500.00
400.00
300.00
200.00
100.00
.00
p = 0.001
Day 0
Day 21
Day 90
p= 0.03
p= 0.01
Nk (abs)
600.00
500.00
400.00
300.00
200.00
100.00
.00
p= 0.04
Day 0
Day 21
Day 90

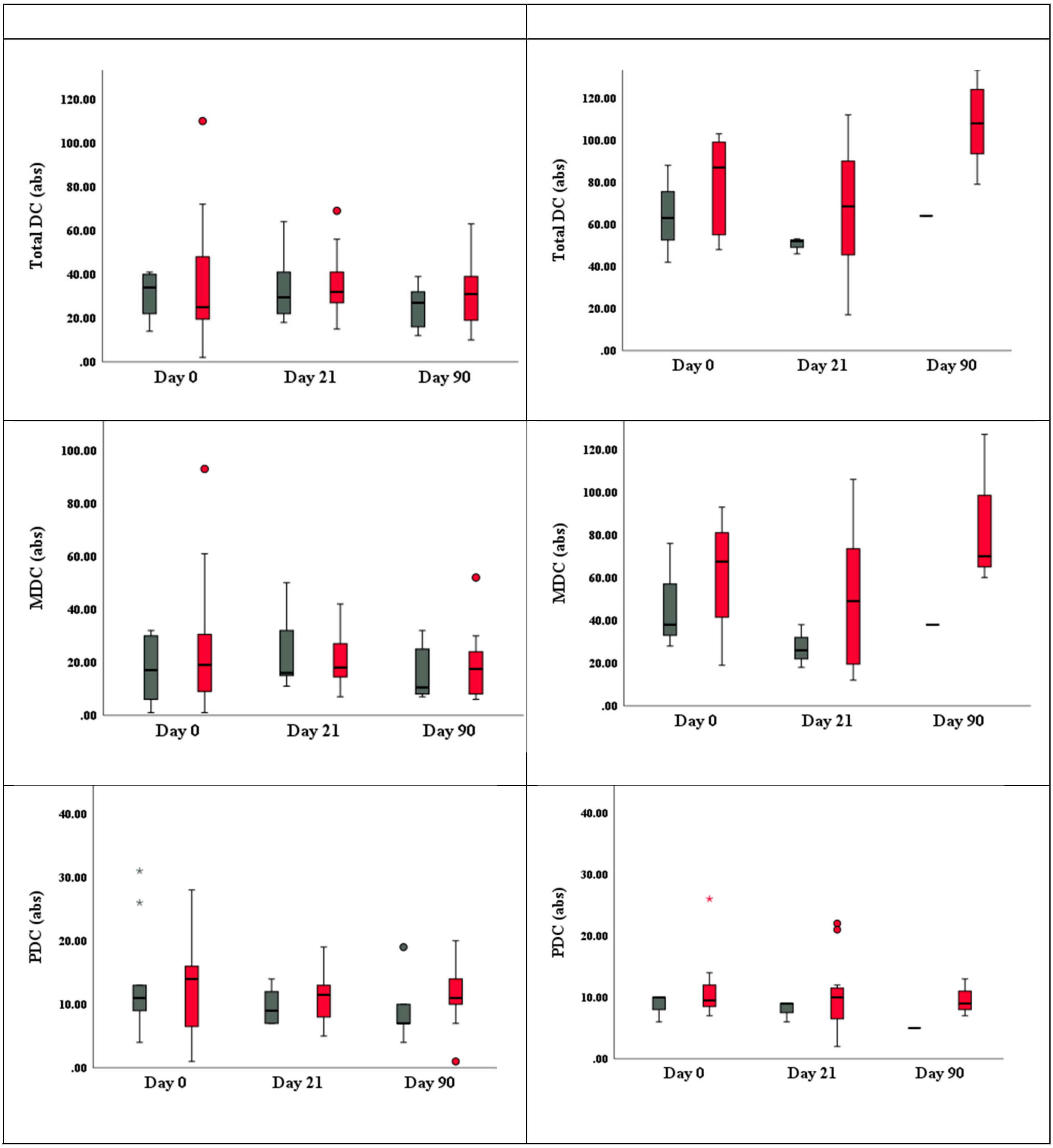

Supplementary Figure S3. Analysis of immune cell counts over time. Percentage (%) and absolute (abs) counts (number of cells × 10^9/L) of different immune cell populations of both cohorts were assessed at different timepoints using flowcytometry. WBC - White blood cells, NK - Natural killer cells, DC - Dendritic cells, MDC - Myeloid dendritic cells, PDC - Plasmacytoid dendritic cells. * denotes p value < 0.05. ** denotes p value < 0.01. Colored dots represent outlier values.
